# Bilateral sequential *Propionibacterium acnes* exogenous endophthalmitis

**DOI:** 10.1186/s12348-016-0084-1

**Published:** 2016-05-24

**Authors:** Norman Saffra, Emily Moriarty, Tatyana Milman

**Affiliations:** Department of Ophthalmology, Maimonides Medical Center, Brooklyn, NY USA; New York Eye and Ear Infirmary, New York, NY USA

**Keywords:** Delayed-onset endophthalmitis, *Propionibacterium acnes*, Pseudophakic endophthalmitis

## Abstract

A 68-year-old man underwent uncomplicated sequential cataract extractions performed more than a year apart. He presented 6 months after the second surgery with persistent intraocular inflammation in both eyes. Cultures from both eyes grew *Propionibacterium acnes* and he responded well to treatment. Suspicion for delayed-onset post-operative endophthalmitis must remain high in uveitis cases that fail to resolve with anti-inflammatory treatments. The authors believe this is the first reported case of bilateral sequential *P. acnes* exogenous endophthalmitis.

## Case report

A 68-year-old main underwent uncomplicated cataract extraction with posterior-chamber intraocular lens (IOL) placement in his right eye in May 2009 and in his left eye in July 2010. Post-operatively, he was tapered off all topical anti-inflammatory medications. In February 2011, he presented with painless vision loss in the left eye and was noted to have marked anterior granulomatous uveitis with mutton-fat keratic precipitates and 3+ anterior chamber cells. Visual acuity at this time was 20/400. The vitreous and right eye examinations were unremarkable. On close follow up, he developed macular edema and optic disc leakage in the left eye. A medical workup by his internist and a rheumatologist, including testing for sarcoidosis, Lyme disease, syphilis, tuberculosis and HLA-B27 antigen were negative. He responded to topical steroids with subsequent improvement in his vision to 20/50. However, in November 2011, he again presented with painless vision loss in the left eye for one week. Acuity at this time was light perception and slit lamp exam reveal an inflamed eye with a 3.5-mm hypopyon. B scan ultrasonography demonstrated an attached retina with vitritis. The patient underwent immediate anterior and posterior tap and injection with ceftazidime and vancomycin followed by a pars plana vitrectomy (PPV) the following day. Aqueous cultures grew *Propionibacterium acnes* sensitive to clindamycin and moxifloxacin. The inflammation resolved following antibiotic treatment and vitrectomy. Final visual acuity was 20/40 and was limited by an epiretinal membrane.

**Fig. 1:**
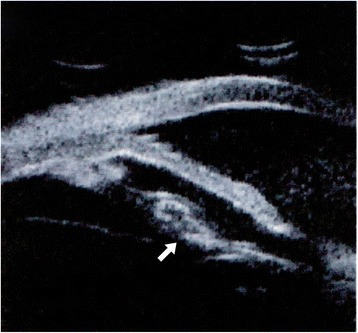
Ultrasound biomicroscopy of the right eye with thickening of the posterior capsule (*arrow*) with an IOL positioned in the capsular bag

**Fig. 2:**
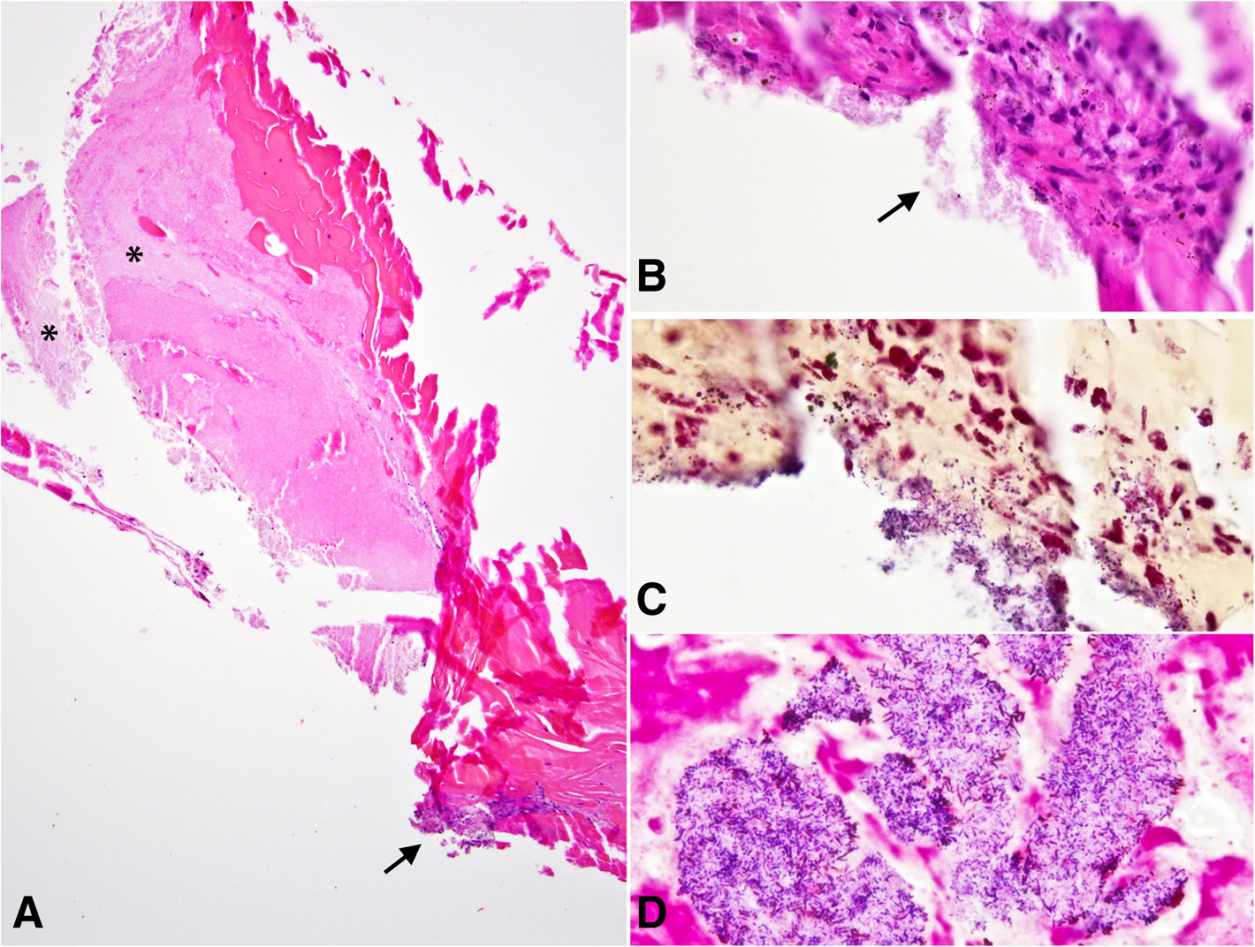
**a**. The explanted lens capsule contains lens cortical material with granular bacterial colonies (*asterisks*), and a sparse focal inflammatory infiltrate. **b**. High-magnification photomicrograph highlights the organisms (*arrow*), associated with a subacute inflammation. **c**. Gram stain confirms the presence of Gram-positive coccobacilli, focally in the cytoplasm of inflammatory cells. **d**. A large colony of Gram-positive pleomorphic coccobacilli is present within the cataractous lens material, unassociated with inflammatory reaction. (stains: hematoxylin-eosin (**a**, **b**) and Gram (**c**, **d**); original magnification ×50 (**a**), ×250 (**b**, **c**, and **d**)

On a subsequent exam in January 2012, the patient was asymptomatic, but noted to have decreased visual acuity in the right eye of 20/60 with granulomatous keratic precipitates with anterior chamber cells. Topical corticosteroids were initiated and the inflammation resolved. Over the next year, he experienced several episodes of recurrent anterior inflammation, each time responding to topical steroids. Repeat blood work and chest x-ray failed to elucidate an etiology. In January 2014, the patient presented with finger counting vision in the right eye, a hypopyon, and elevated intraocular pressure. PPV with injection of antibiotics was performed and vitreous cultures grew *P. acnes* sensitive to clindamycin. The hypopyon recurred after cessation of treatment and ultrasound biomicroscopy of the anterior chamber demonstrated thickening of the peripheral lens capsule encapsulating the posterior chamber intraocular lens [Fig. 1]. The patient underwent PPV with explantation of the IOL, capsulectomy, and injection of intravitreal clindamycin. Histopathologic examination of the capsule confirmed the presence of *P. acnes*. [Figure 2] Following a 10-month period of aphakia without recurrent inflammation, the patient had a secondary anterior chamber IOL placed and achieved a final visual acuity of 20/25 in the right eye.

## Discussion

Chronic post-operative endophthalmitis (CPE) is characterized as an infectious intraocular inflammation that occurs more than 6 weeks after ocular surgery—sometimes months to years later. It can mimic other types of ocular inflammation and may respond initially to corticosteroid treatment. Cases of chronic post-operative inflammation that fail to resolve with corticosteroids should elicit a high suspicion for CPE.

*P. acnes* is an anaerobic, gram-positive bacillus that is found as part of the normal flora of the skin, hair follicles and conjunctiva [1]. It has been identified as the causative agent for various implant-associated infections and accounts for the majority of CPE cases [2]. Formation of a posterior capsular plaque is the most classic finding of *P. acnes* CPE; however, it is not always present. The small number and low virulence of the organism often result in a low yield in microscopy and negative culture from anterior chamber (AC) taps. [3]. Pars plana vitrectomy and/or polymerase chain reaction may improve detection in cases where a high suspicion is maintained despite negative AC cultures.

While bilateral *P. acnes* endogenous endophthalmitis presenting as scleritis and uveitis has been reported in a patient who did not undergo ocular surgery [4], we believe this is the first report of exogenous bilateral sequential *P. acnes* post-operative endophthalmitis. This case highlights the importance of maintaining a high suspicion of *P. acnes* endophthalmitis in a post-operative patient with chronic inflammation. PPV with IOL explantation, capsulectomy, and intravitreal antibiotics results in the lowest recurrence rate; however, this may not be necessary in all cases, and treatment should be tailored for each patient.

## Abbreviations

AC, anterior chamber; CPE, chronic post-operative endophthalmitis; IOL, intraocular lens; P. acnes; Propionibacterium acnes; PPV pars plana vitrectomy
